# Identification of a Novel *DNAAF3* Variant in a 54‐Year‐Old Patient With Newly Diagnosed Primary Ciliary Dyskinesia (PCD)

**DOI:** 10.1155/crig/5137651

**Published:** 2026-01-06

**Authors:** Mirja M. Wirtz, Sabine Ebner, Anna Pleyers, Natalie Firlei-Fleischmann, Richard Untersteiner, Michael Studnicka

**Affiliations:** ^1^ Department of Pneumology, Salzburger Landeskliniken, Paracelsus Medical University, Salzburg, Austria, pmu.ac.at; ^2^ Institute of Human Genetics, Salzburger Landeskliniken, Paracelsus Medical University, Salzburg, Austria, pmu.ac.at

## Abstract

Primary ciliary dyskinesia (PCD) is a rare and heterogeneous inherited disease characterized by impaired mucociliary clearance. Patients with PCD typically present with recurrent respiratory infections resulting in the development of bronchiectasis. Even though awareness of the disease has increased over the years, PCD remains underdiagnosed. We here present a case of a newly diagnosed middle‐aged female found to have a previously undescribed variant of the disease‐associated *DNAAF3* gene.

## 1. Introduction

Primary ciliary dyskinesia (PCD) encompasses a rare spectrum of inherited diseases all characterized by an impaired ciliary function [1, 2]. As mucociliary clearance is consequently compromised, most patients with PCD present with symptoms of chronic upper and lower respiratory infections [3]. However, due to the genetic heterogeneity of the disease with an association of at least 50 genes, phenotypic presentation of patients with PCD is considerably variable further hindering establishment of the correct diagnosis [1]. Whereas most patients with PCD are diagnosed at a young age [4], we here present a new diagnosis of PCD in an adult patient, who was furthermore found to have a novel pathogenic variant of the Dynein Axonemal Assembly Factor 3 (*DNAAF3)* gene. *DNAAF3* is involved in the assembly of the outer and inner dynein arm complexes and therefore in ciliary motility [1, 5].

## 2. Case Description

**Figure 1:**
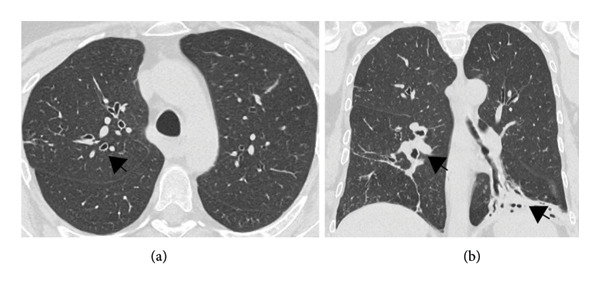
High‐resolution computed tomography (HRCT) with axial (a) and coronal (b) views showing bronchiectasis and cystic changes in consolidations (see black arrows).

A 54‐year‐old female was referred to the pulmonary outpatient clinic of our tertiary care center in Salzburg, Austria, for further evaluation of a chronic productive cough, recurrent pulmonary infections, and a thoracic computed tomography (CT) scan showing bronchiectasis and consolidations with cystic changes (Figure 1).

On detailed history taking, the patient, who was an only child, reported an uncomplicated birth without neonatal respiratory distress or hospitalization and a history of recurrent middle ear and chronic sinus infections since her early childhood. Unaware of the specific circumstances, the patient additionally reported to have needed a middle lobe resection at the age of 9, to have required in vitro–fertilization to become pregnant with each of her children, and to have a positive family history for pulmonary diseases. Other comorbidities were limited to impaired hearing with a necessity for hearing aids and arterial hypertension.

Except for a nasal voice, physical examination was unremarkable.

Prompted by the conspicuous medical history and the existing CT findings, which showed no evidence of organ laterality anomalies, routine diagnostic work‐up—including pulmonary function (showing a nonspecific ventilatory pattern and a mildly reduced diffusion capacity of 68.4%), broad laboratory (incorporating serology), and microbiological (sputum culture) testing—was expanded by nasal nitric oxide and sweat chloride testing.

Against the background of unremarkable laboratory tests, a physiological sweat chloride test (11 mmol/L), a positive sputum culture for *Haemophilus influenzae*, and an abnormally low nasal nitric oxide level of 43 parts per billion (ppb), molecular genetic testing for PCD and cystic fibrosis was ultimately performed.

**Figure 2:**
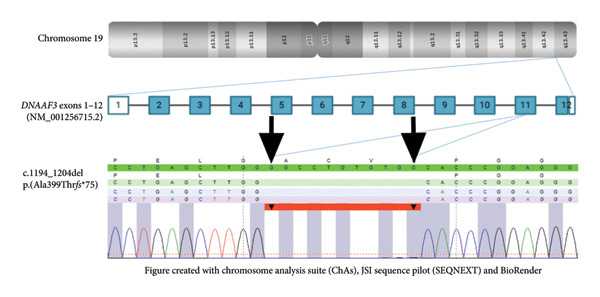
Overview of the *DNAAF3* gene and the patient’s variant with black arrows indicating the beginning and the end of the deletion.

Genomic DNA isolated from a peripheral blood sample of the patient was analyzed by whole‐exome sequencing and revealed detection of the novel, homozygous, and likely pathogenic variant NM_001256715.2:c.1194_1204del of the *DNAAF3* gene (Figure 2). The variant is located in Exon 11 (total gene exons: 12) and encompasses the removal of 11 nucleotides (GGCCTGTGTGG). Follow‐up questioning did not reveal consanguinity of the patient’s parents, who furthermore did not originate from the same region.

In addition to genetic counseling, the patient was started on airway clearance therapy and supportive care. Since her diagnosis 9 months ago, the patient did not suffer from an acute exacerbation of her disease.

## 3. Discussion

To the best of our knowledge, this is the first reported case describing PCD with the detected *DNAAF3*‐variant c.1194_1204del, a frameshift mutation most likely resulting in a nonfunctioning protein due to a premature stop codon after 74 altered amino acids (p.(Ala399Thr*fs*∗75)).

Our detected variant is reported at a frequency of 0.0001% in the population database gnomAD (v4.1.0) [6]. Although the database ClinVar does not contain such an entry, it lists a pathogenic variant with an alternate exchange at the same amino acid position (p.(Ala399Pro*fs*∗9)) [7]. Given these circumstances, we classify our newly identified *DNAAF3*‐variant c.1194_1204del as a novel likely pathogenic variant associated with PCD.

*DNAAF3* is a gene on Chromosome 19q13 fundamental in the initial assembly of the dynein arms that generate ciliary motion. Biallelic mutations within the *DNAAF3* gene are linked to PCD‐2 (CILD2, OMIM #606763), a disease characterized by nonfunctioning motile cilia due to absent outer and inner dynein arms [8]. This gene has been firstly associated with PCD in 2012 and is one of the 50 genes that are nowadays known to be mutated in PCD. As most other genes linked to PCD, it is inherited in an autosomal recessive manner [1, 5].

Respiratory manifestations of PCD‐2 usually present within the first year of life; other clinical features include bronchiectasis, otitis media, nasal polyps, hearing loss, infertility and situs inversus [5, 8].

Even though our adult patient has exhibited most of the typical features of PCD for decades, her diagnosis was not established until reaching age 54. This diagnostic delay highlights the complexity diagnosing PCD and is, unfortunately, not uncommon—provided the disease is identified at all [1, 4, 9, 10].

## Consent

No written consent has been obtained from the patient as there are no patient identifiable data included in this case report.

## Conflicts of Interest

The authors declare no conflicts of interest.

## Funding

No funding was received for this manuscript.

## Data Availability

The research data are not shared.
